# Supplementary material for: Synthesis and PET Imaging Biodistribution Studies of Radiolabeled Iododiflunisal, a Transthyretin Tetramer Stabilizer, Candidate Drug for Alzheimer’s Disease
Source: Molecules. 2024 Jan 18;29(2):488. doi: 10.3390/molecules29020488 (PMC10818730; doi:10.3390/molecules29020488)
Supplement: Supplementary file 1 [file molecules-29-00488-s001.zip › molecules-2767398-supplementary.pdf]

Synthesis and PET imaging biodistribution studies of radiolabeled iododiflunisal, a transthyretin tetramer stabilizer, candidate drug for Alzheimer's disease

Sameer M. Joshi,[1,2] Thomas Wilson,[3] Zibo Li,[2,4] Sean Preshlock,[3] Vanessa Gómez Vallejo,[1] Véronique Gouverneur,[3] Jordi Llop[1,*] and Gemma Arsequell[5,*]

[1] CIC biomaGUNE, Basque Research and Technology Alliance (BRTA), Paseo Miramón 182, Parque Tecnológico de San Sebastián, 20009 Donostia-San Sebastián, Gipuzkoa, Spain; S.M.J.: sameerjoshi94@gmail.com; V.G.-V.: vgomez@cicbiomagune.es; J.L.: jllop@cicbiomagune.es

[2] Department of Radiology, The University of North Carolina at Chapel Hill, Chapel Hill, North Carolina 27599, United States. sameerjoshi94@gmail.com

[3] Chemistry Research Laboratory, Oxford University, OX1 3TA, UK. T.W.: tcwilson1610@gmail.com; V.G.: S.P. deceased; V.G.: veronique.gouverneur@chem.ox.ac.uk

[4] Lineberger Comprehensive Cancer Center, The University of North Carolina at Chapel Hill, Chapel Hill, North Carolina 27514, United States. zibo_li@med.unc.edu

[5] Institut de Química Avançada de Catalunya (IQAC), Spanish National Research Council (CSIC), 08034, Barcelona, Spain. gemma.arsequell@iqac.csic.es

***** Correspondence: J.L.: jllop@cicbiomagune.es; G.A. gemma.arsequell@iqac.csic.es.

^{1}H, ^{13}C, and ^{19}F NMR Data:

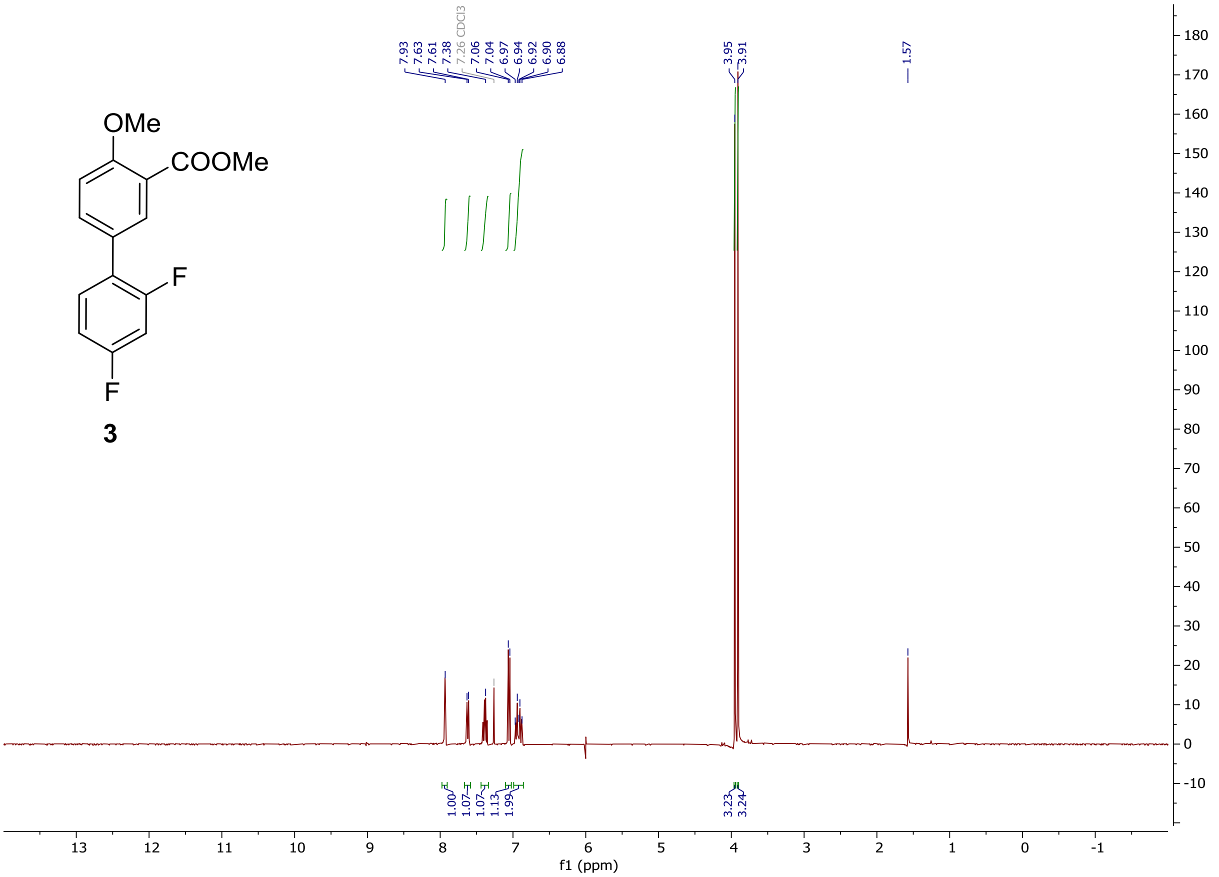

Figure S1: ^{1}H NMR of compound **3** at 400 MHz NMR spectrometer.

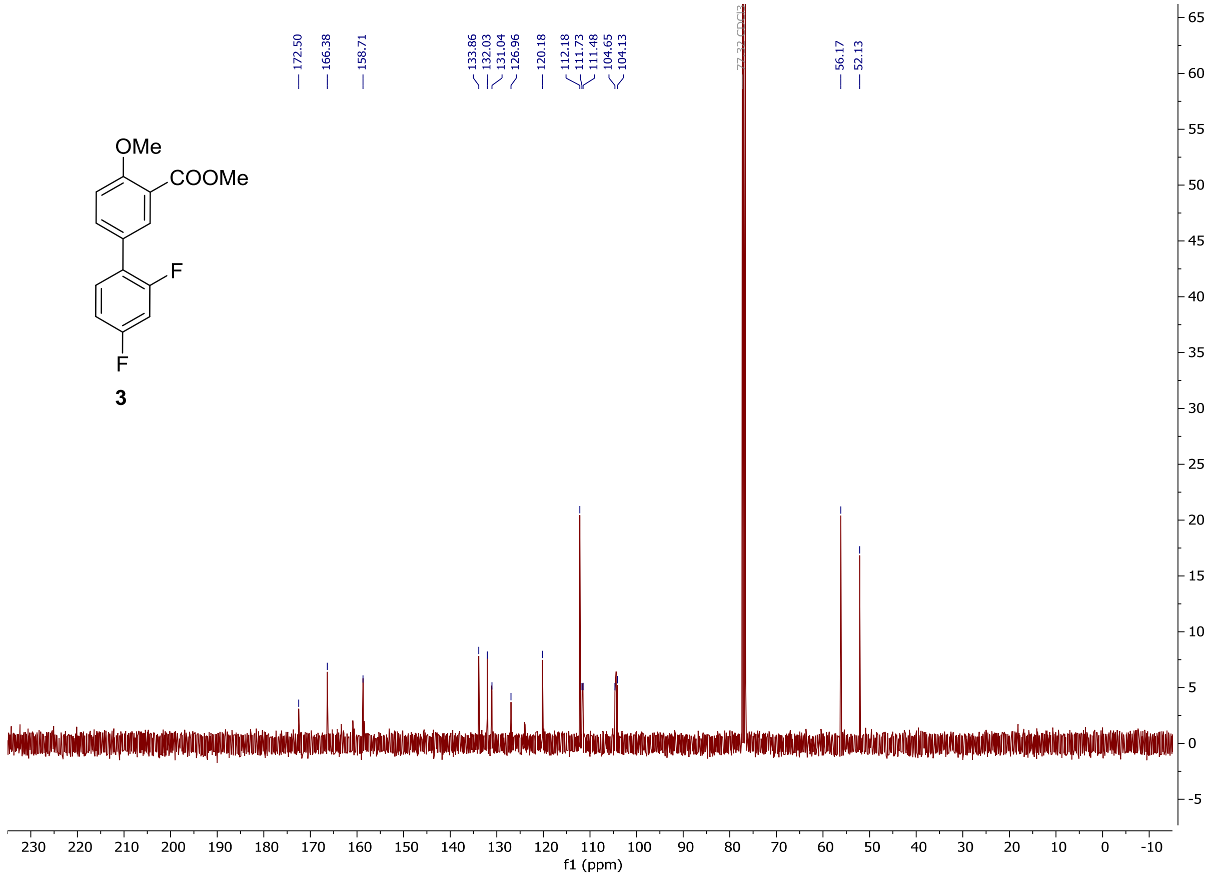

Figure S2: ^{13}C NMR of compound **3** at 100 MHz NMR spectrometer.

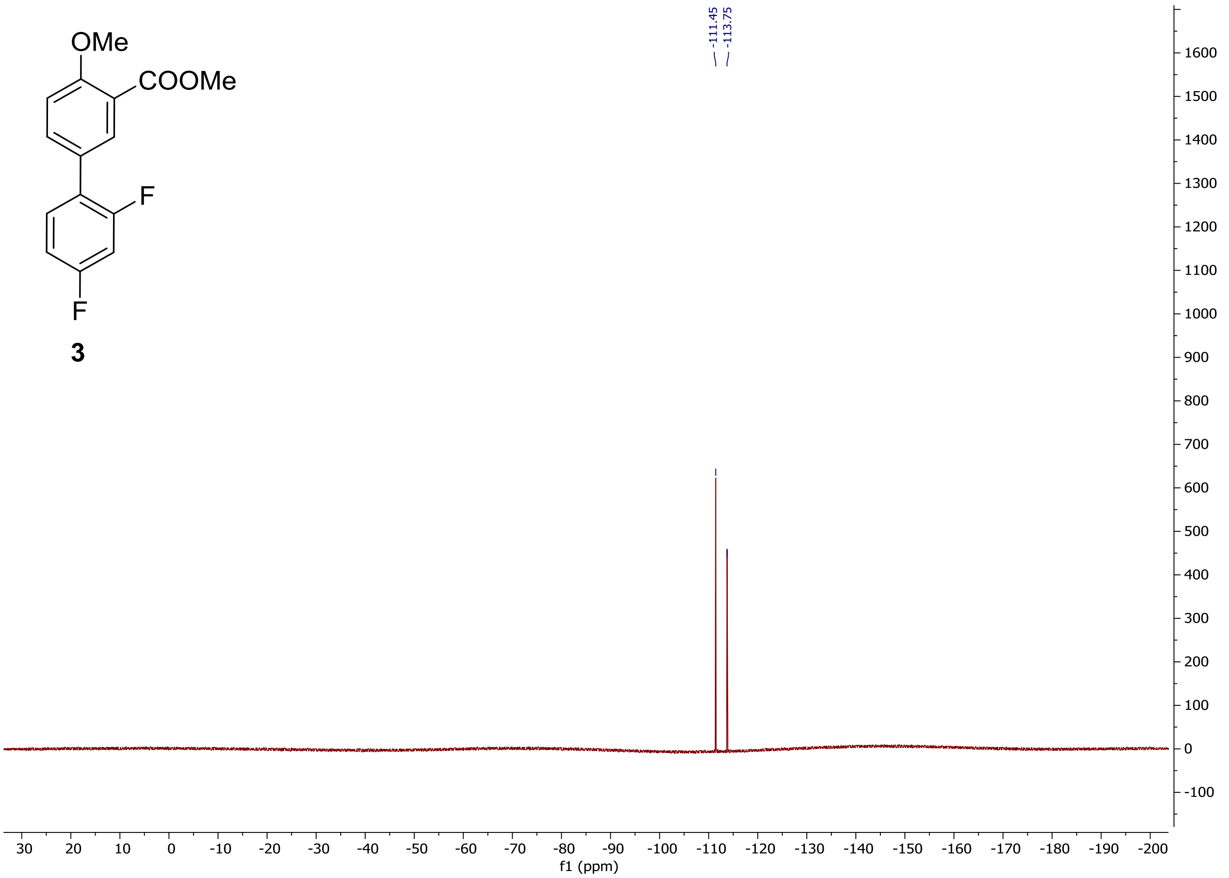

Figure S3: ^{19}F NMR spectrum of methyl compound **3** at 400 MHz NMR spectrometer.

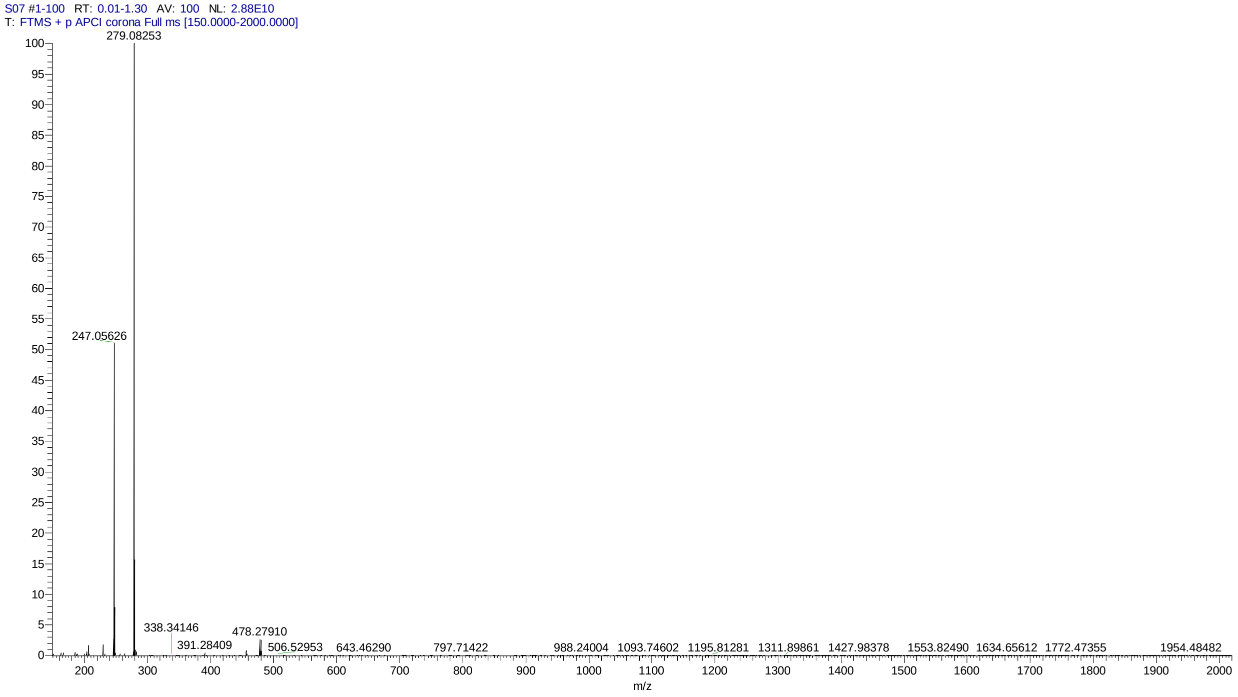

Figure S4: HRMS spectrum of methyl compound **3**.

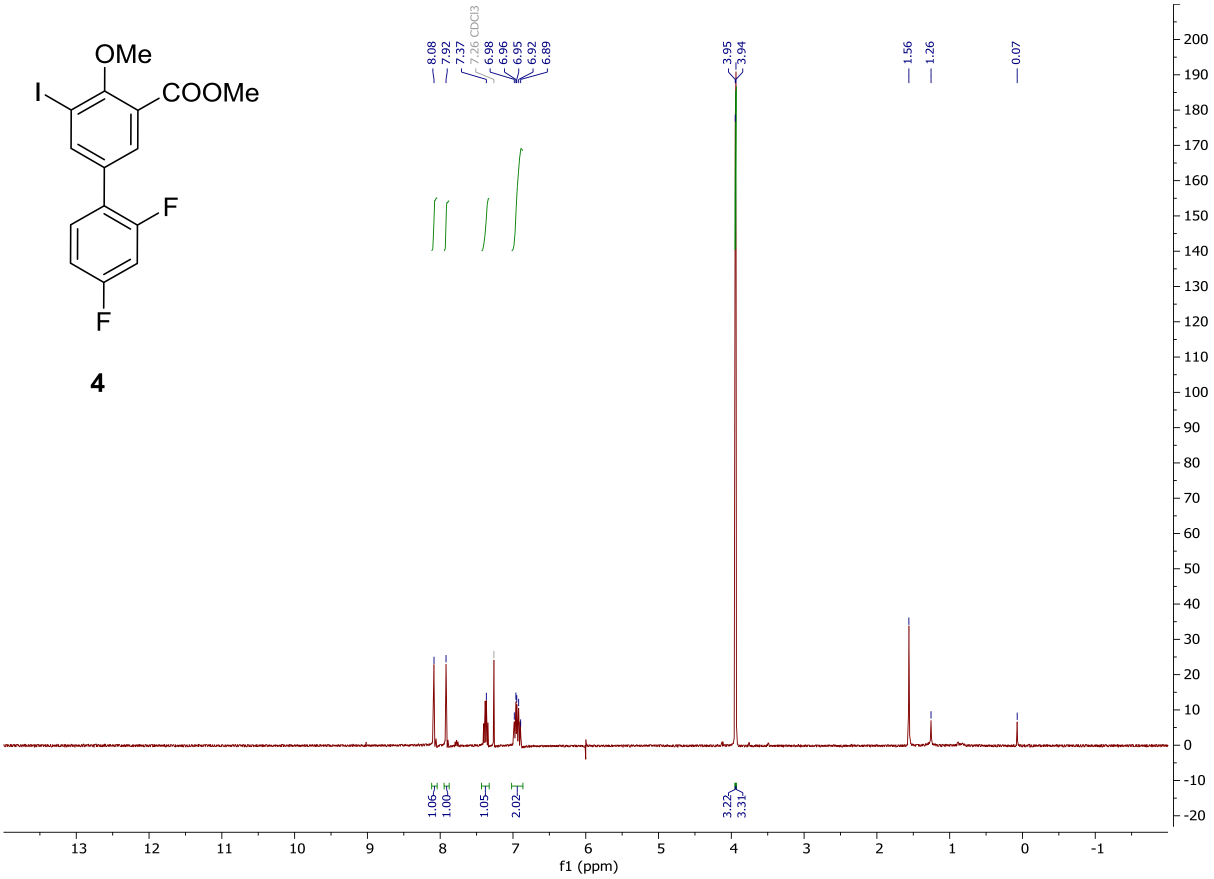

Figure S5: ^{1}H NMR spectrum of compound **4** at 400 MHz NMR spectrometer.

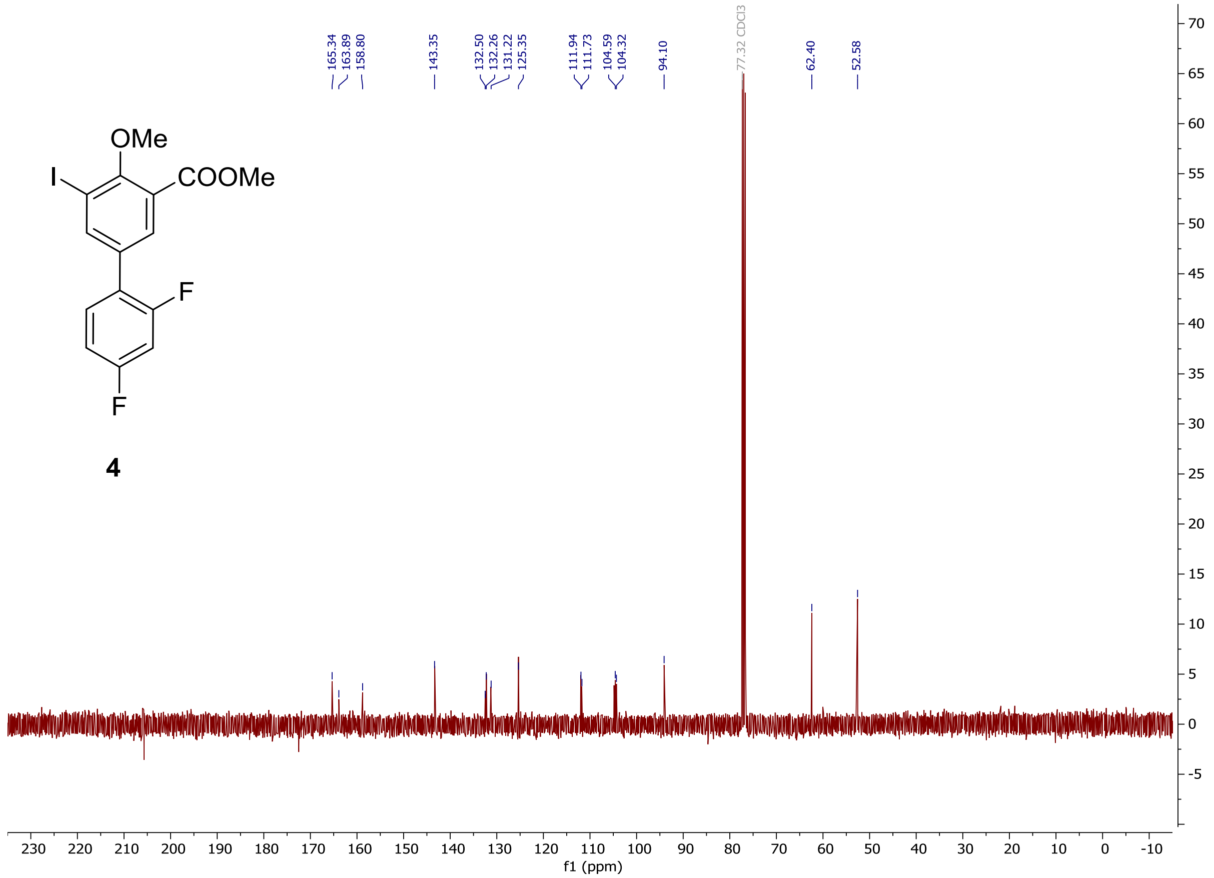

Figure S6: ^{13}C NMR spectrum of compound **4** at 100 MHz NMR spectrometer.

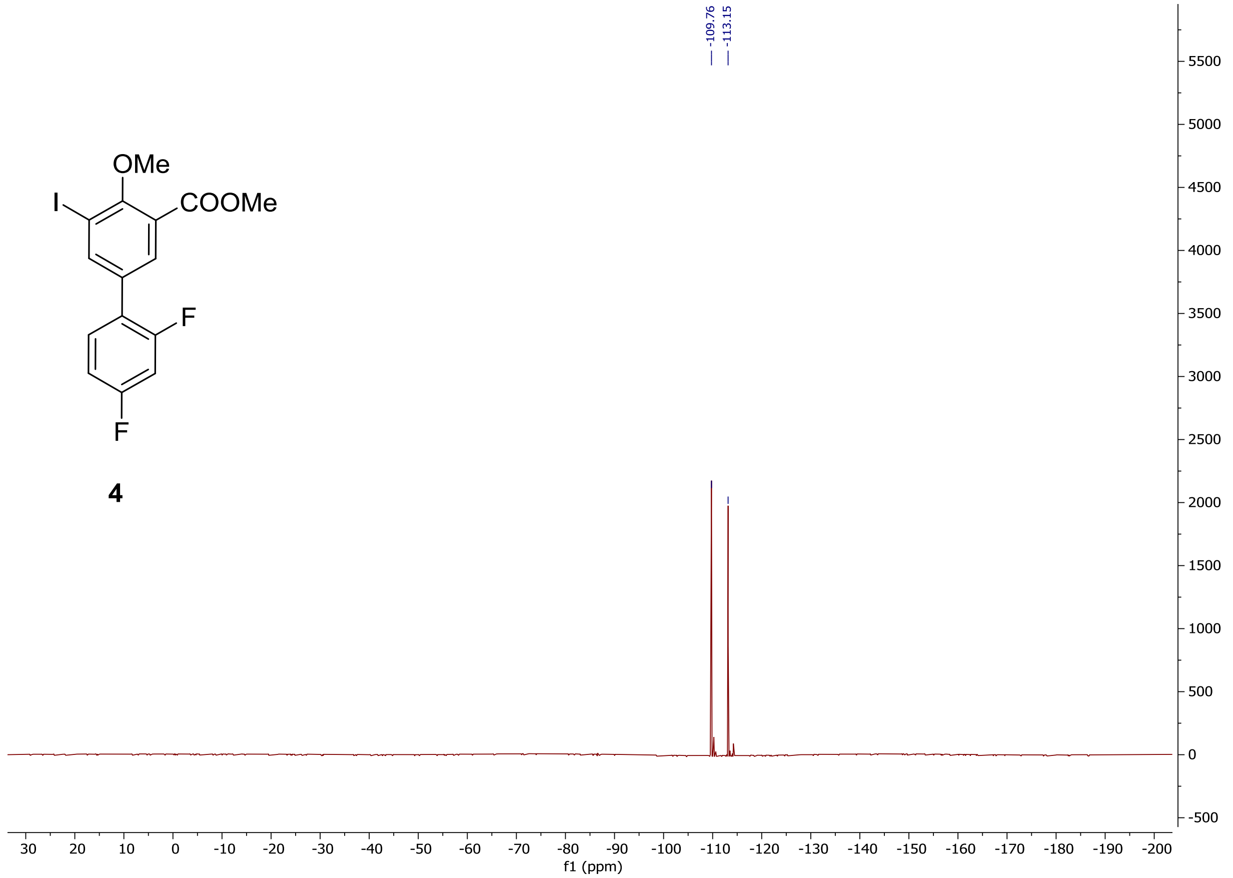

Figure S7: ^{19}F NMR spectrum of methyl compound **4** at 400 MHz NMR spectrometer.

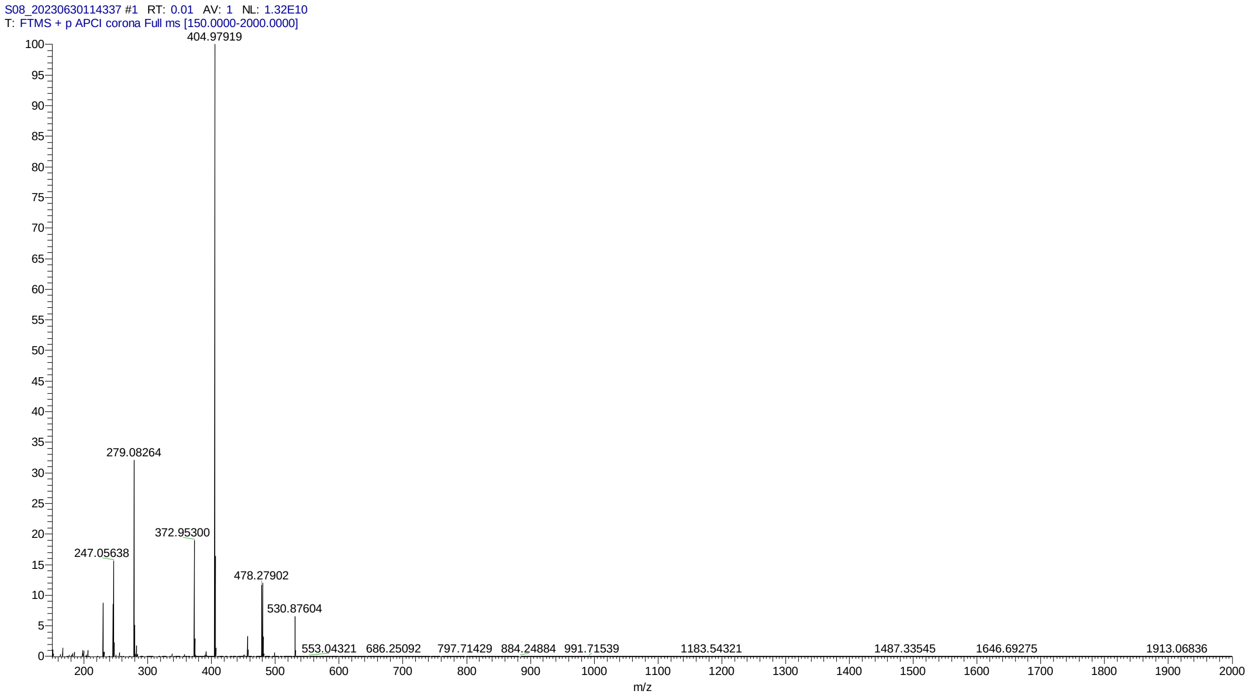

Figure S8: HRMS spectrum of compound **4**.

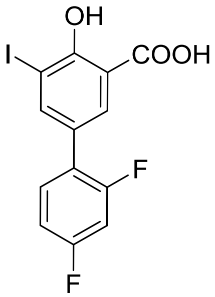

IDIF

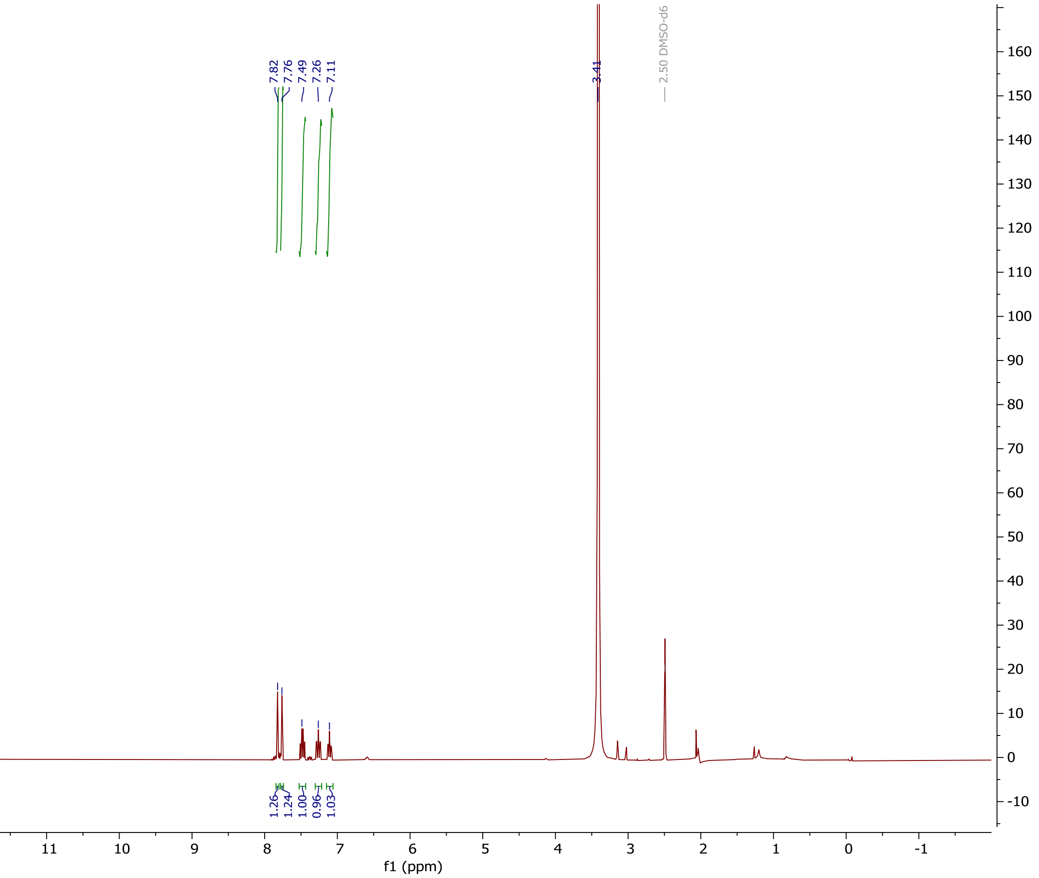

Figure S9: ^{1}H NMR spectrum of IDIF **2** at 400 MHz NMR spectrometer.

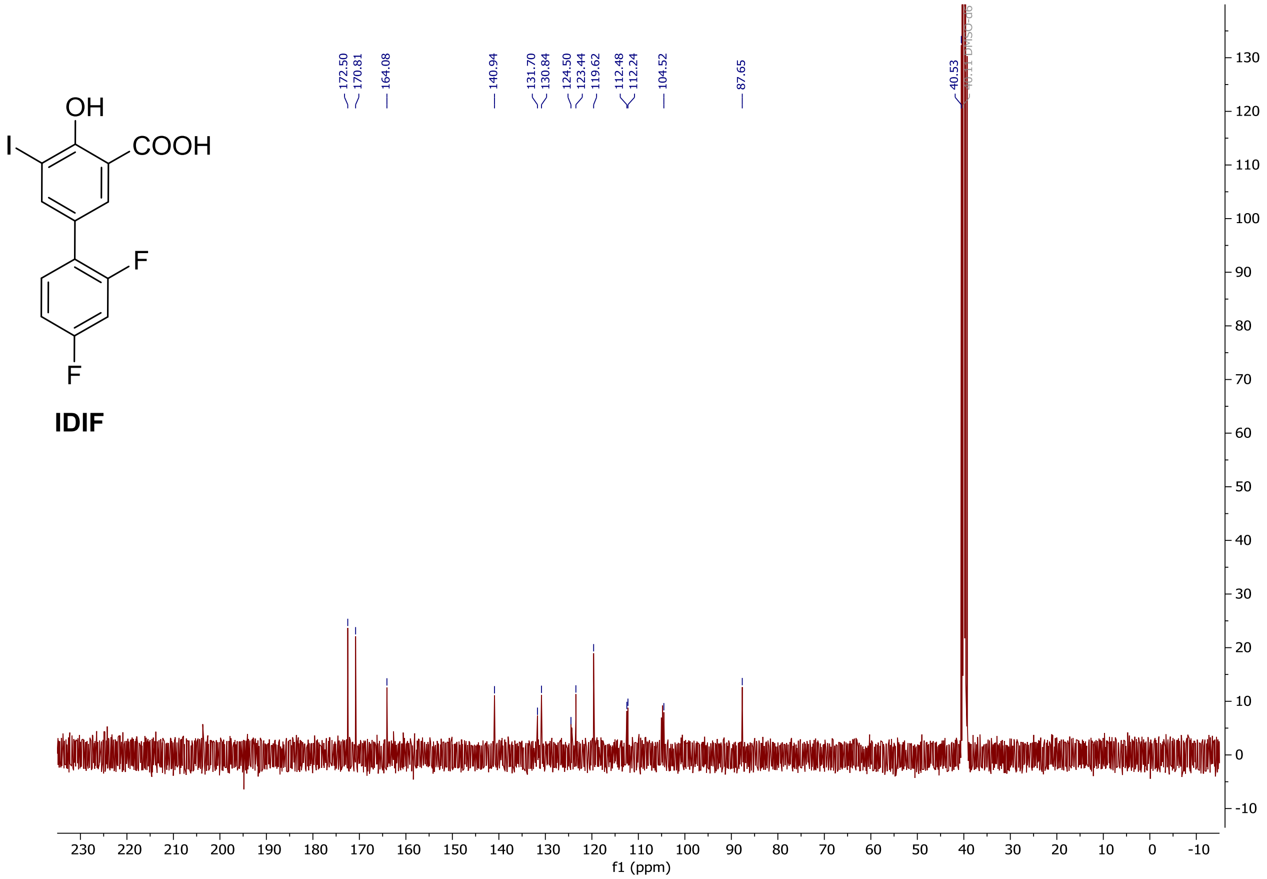

Figure S10: ^{13}C NMR spectrum of IDIF **2** at 100 MHz NMR spectrometer.

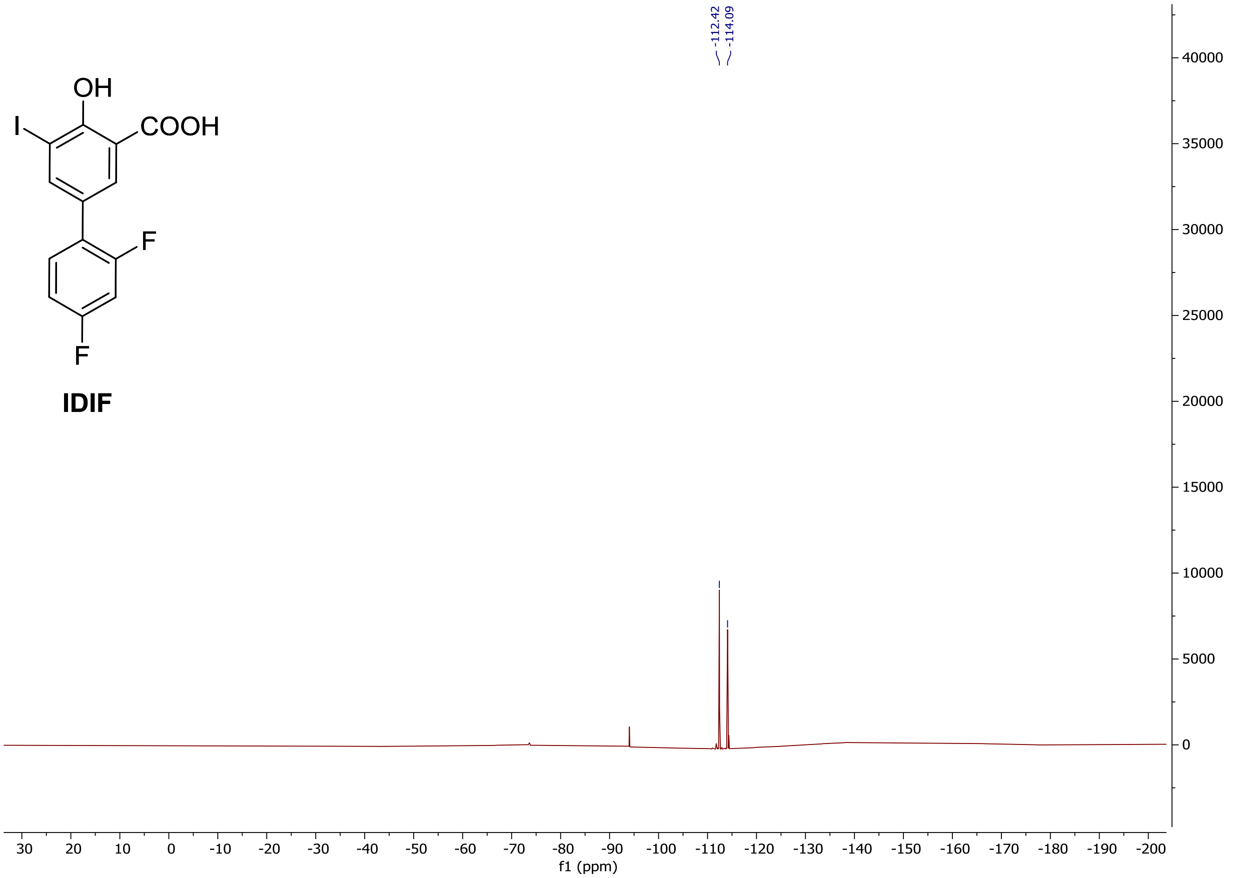

Figure S11: ^{19}F NMR spectrum of IDIF **2** at 400 MHz NMR spectrometer.

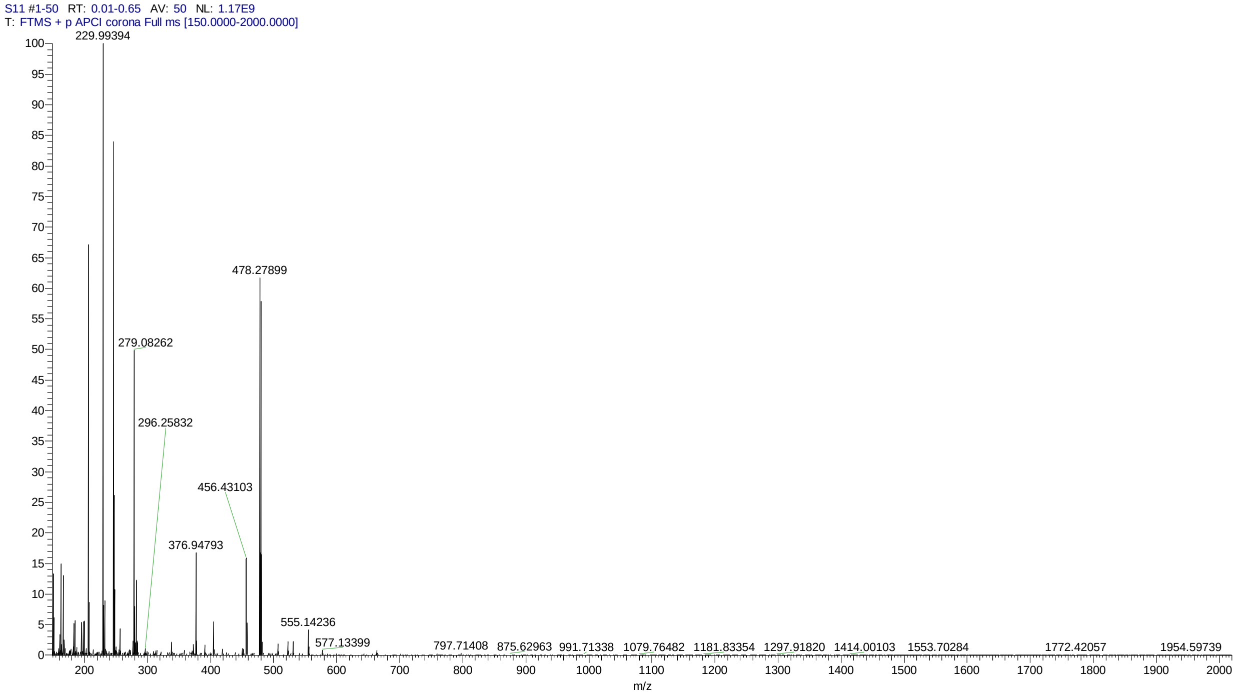

Figure S12: HRMS spectrum of IDIF **2**.

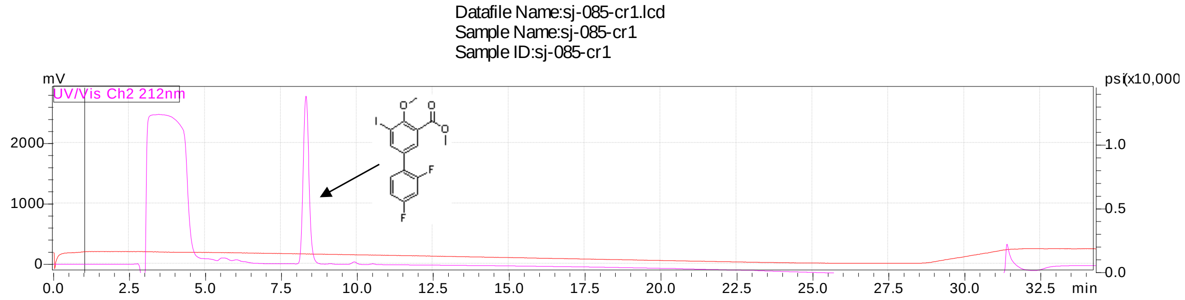

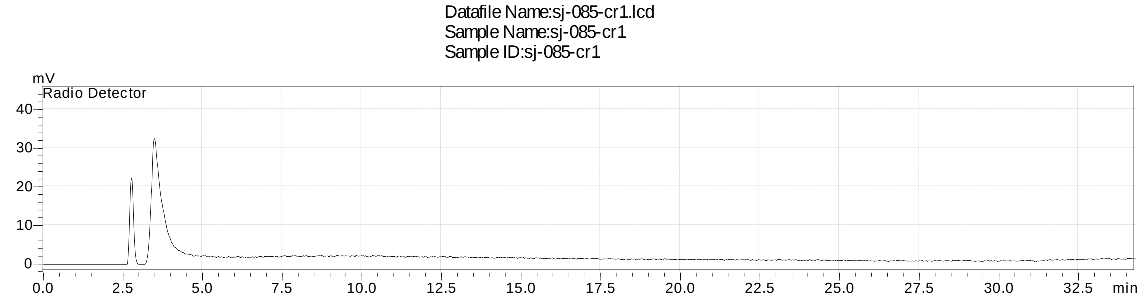

Figure S13: HPLC chromatogram (UV-212 nm and radioactive detector) for ^{18}F exchange study in NMP solvent at 140^{0}C for 30 minutes. No peaks are detected in the radioactive chromatogram at retention time = 8-8.5 min, confirming that isotopic exchange reaction did not occur under these experimental conditions.

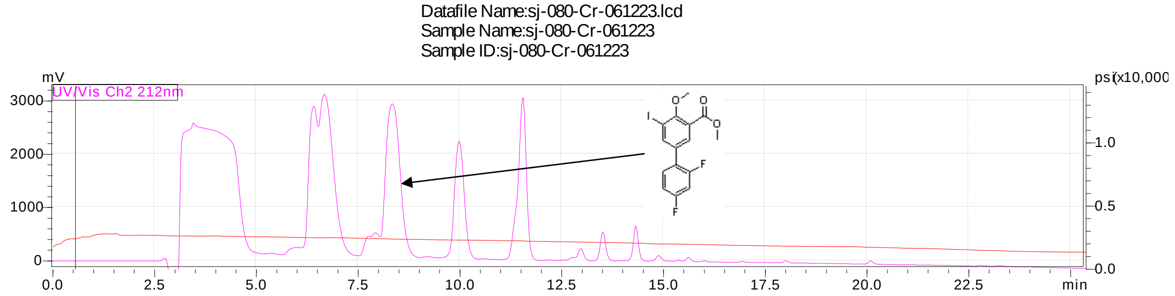

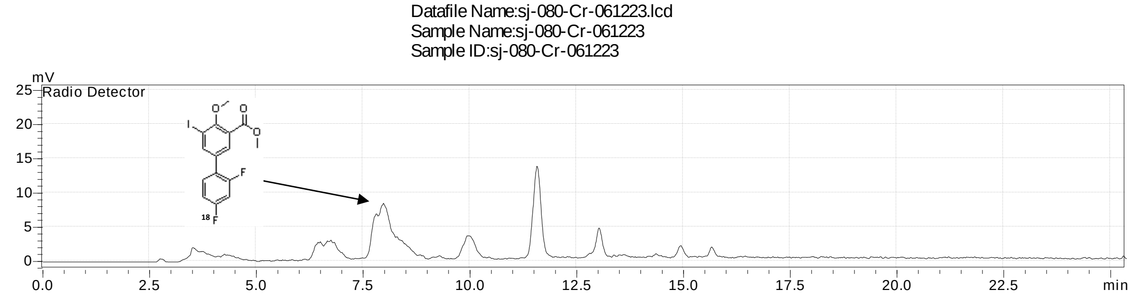

Figure S14: HPLC chromatogram (UV-212 nm and radioactive detector) for ^{18}F exchange study in NMP solvent at 180^{0}C for 30 minutes. The presence of a peak at the same retention time as the reference standard (retention time = 8-8.5 min) suggests the formation of the desired labelled compound, although several labelled impurities are also observed.

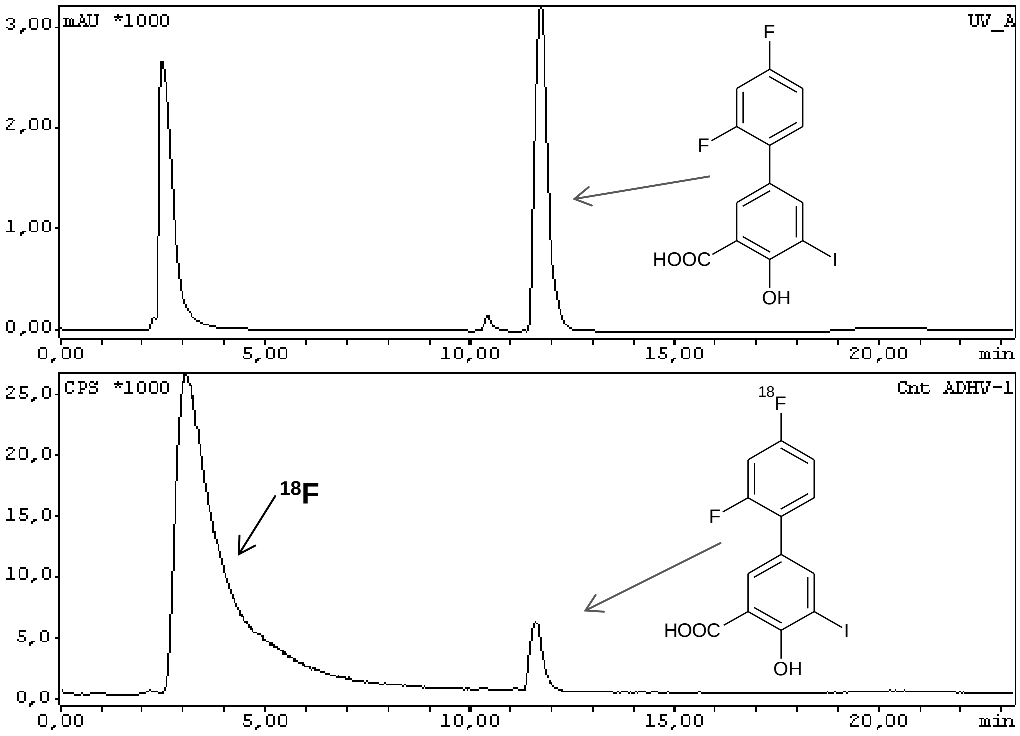

Figure S15: HPLC chromatogram (UV-254 nm and radioactive detector) for ^{18}F exchange study in DMSO solvent at 160^{0}C for 20 minutes. The presence of a peak at the same retention time as the reference standard suggests the formation of the desired labelled compound.

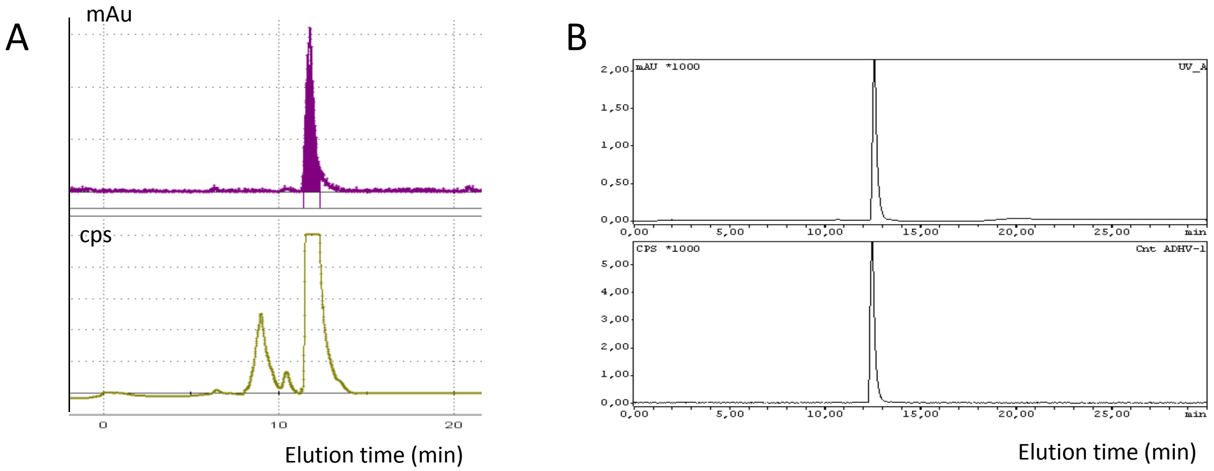

Figure S16: A) Semi-preparative HPLC chromatogram (UV-254 nm and radioactive detector) for ^{18}F exchange study in DMSO solvent at 160^{0}C for 20 minutes. The collected peak (retention time ca. 12 min) is indicated with solid filling; B) HPLC chromatogram (UV-254 nm and radioactive detector) corresponding to quality control of the collected fraction.
